# Supplementary material for: Protein or ribonucleoprotein-mediated blocking of recombinase polymerase amplification enables the discrimination of nucleotide and epigenetic differences between cell populations
Source: Commun Biol. 2021 Aug 19;4:988. doi: 10.1038/s42003-021-02503-5 (PMC8376914; doi:10.1038/s42003-021-02503-5)
Supplement: Supplementary file 2 — Supplementary Information [file 42003_2021_2503_MOESM2_ESM.pdf]

Supplementary Information

Protein or ribonucleoprotein-mediated blocking of recombinase polymerase amplification enables the discrimination of nucleotide and epigenetic differences between cell populations

Toshitsugu Fujita, Shoko Nagata, Hodaka Fujii

Supplementary Table 1. Oligonucleotides used in this study

Categories	Name	Sequence (5' → 3')	Experiments	Number
ORN	ORN_KRAS	guggcguaggcaagagugc	Figure 2c–2e, and 2g, and Supplementary Figure 1	-
ORN	ORN_KRAS_mut	gucgcguaggcaagagugc	Figure 2d and 2e	-
ORN	ORN_KRAS#2	cugguggcguaggcaagag	Figure 2g	-
ORN	ORN_KRAS#3	guaguuggagcugguggcgu	Supplementary Figure 2b and 2c	-
ORN	ORN_p16	gcggcccggggucggguaga	Figure 2c	-
ORN	ORN_cPax5_Ex1B	cgacccguuugcagcaaugc	Figure 2c	-
crRNA	gRNA_KRAS	guaguuggagcugguggcguguuuuagagcuaugcuguuuug	Figure 3b and 3c, and Supplementary Figures 3c, 4d, and 5b	-
crRNA	gRNA_KRAS#2	cuugugguaguuggagcuggguuuuagagcuaugcuguuuug	Figure 3b and 3c, and Supplementary Figure 6a and 6c	-
crRNA	gRNA_KRAS#3	aaacuugugguaguuggagcguuuuagagcuaugcuguuuug	Supplementary Figure 4b	-
crRNA	gRNA_KRAS_mut	guaguuggagcuggaggcguguuuuagagcuaugcuguuuug	Figure 3e and Supplementary Figure 4d	-
crRNA	gRNA_hp16_Gx5#2	acggccgcggcccgggggucguuuuagagcuaugcuguuuug	Figure 3b and 3c, and Supplementary Figures 4d and 5b	-
crRNA	gRNA_mid2	caccuccucuacccgaccccguuuuagagcuaugcuguuu	Figure 3i	-
tracrRNA	-	aaacagcauagcaaguuaaaauaaggcuaguccguuaucaacuugaaaaaguggcaccgagucggugcu	Figure 3b, 3c, 3e, 3i, and Supplementary Figures 3c, 4b, 4d, 5b, 6a, and 6c	-
ODN_2'3'ddC	ODN_KRAS_ddC	tcttgcctacgccaccagctccaac	Supplementary Figure 12c and 12d	28759
Primer	KRAS-RPA-G12-F	tagtgtattaaccttatgtgtgacatgttctaat	Figures 2c–e, 2g, 3b, 3c, and 3e, and Supplementary Figures 1, 2b, 2c, 3b, 3c, 4b, 4d, 6a, 6c, and 8d	28318
Primer	KRAS-RPA-G12-R	aaacaagatttacctctattgttggatcatattc	Figures 2c–e, 2g, 3b, 3c, and 3e, and Supplementary Figures 1, 2b, 2c, 3b, 3c, 4b, 4d, 6a, 6c, and 8d	28319
Primer	hKRAS-G12G13-F	ggtgagtttgtattaaaaggtactgg	Supplementary Figure 12c and 12d	28685
Primer	hKRAS_G12G13_antisence_clamp	ttagctgtatcgtcaaggcactc	Supplementary Figure 12c and 12d	28760
Primer	p16-RPA-F2	ggcggcggggagcagcatggagccttcggctgac	Figure 3i, and Supplementary Figures 5b and 7a	28315
Primer	p16-RPA-R2	ctacccacctggatcggcctccgaccgtaactat	Figure 3i, and Supplementary Figures 5b and 7a	28316
Primer	p16-RPA-F	gaggaagaaagaggaggggctggctggtcacc	Supplementary Figure 10b and 10d	28313
Primer	p16-RPA-R	ctgcagaccctctacccacctggatcggcctc	Supplementary Figure 10b	28314
Primer	hCDKN2A-(-)Bisul-R2	tgcagaccctctacccacctggat	Supplementary Figure 10d	27477
Primer	p14-RPA-F	gtcccagtctgcagttaagggggcaggagt	Figure 4c and 4f, and Supplementary Figure 9a	28354
Primer	p14-RPA-R	gggcctttcctacctggtcttctaggaa	Figure 4c and 4f, and Supplementary Figure 9a	28355
Primer	Pax5-LexA-RPA-F	gcatcagtcgcccttcgcctccttctctcg	Supplementary Figure 8b and 8c	28467
Primer	Pax5-LexA-RPA-R	gcgagggcggaacgtgactttgccctgcgg	Supplementary Figure 8b and 8c	28468
Primer	EGFR-RPA-L858-F	tggcagccaggaatgtactggtgaaaacactgcagcatg	Supplementary Figure 13d	28364
Primer	EGFR-RPA-L858-R3	cagaatgtctggagagcatcctcccctgcatg	Supplementary Figure 13d	28368

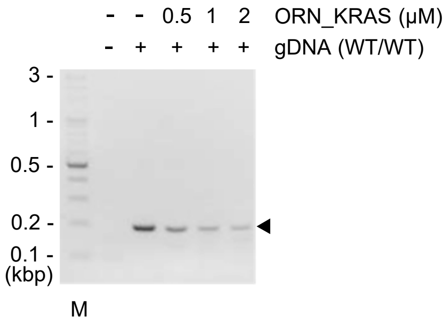

Supplementary Figure 1. ORNi-RPA with various doses of an ORN. The human *KRAS* gene was amplified from 293T gDNA (WT/WT *KRAS*) in the presence of various doses of ORN_KRAS. 0.5–2 μM of the ORN moderately suppressed *KRAS* amplification. M, molecular weight marker.

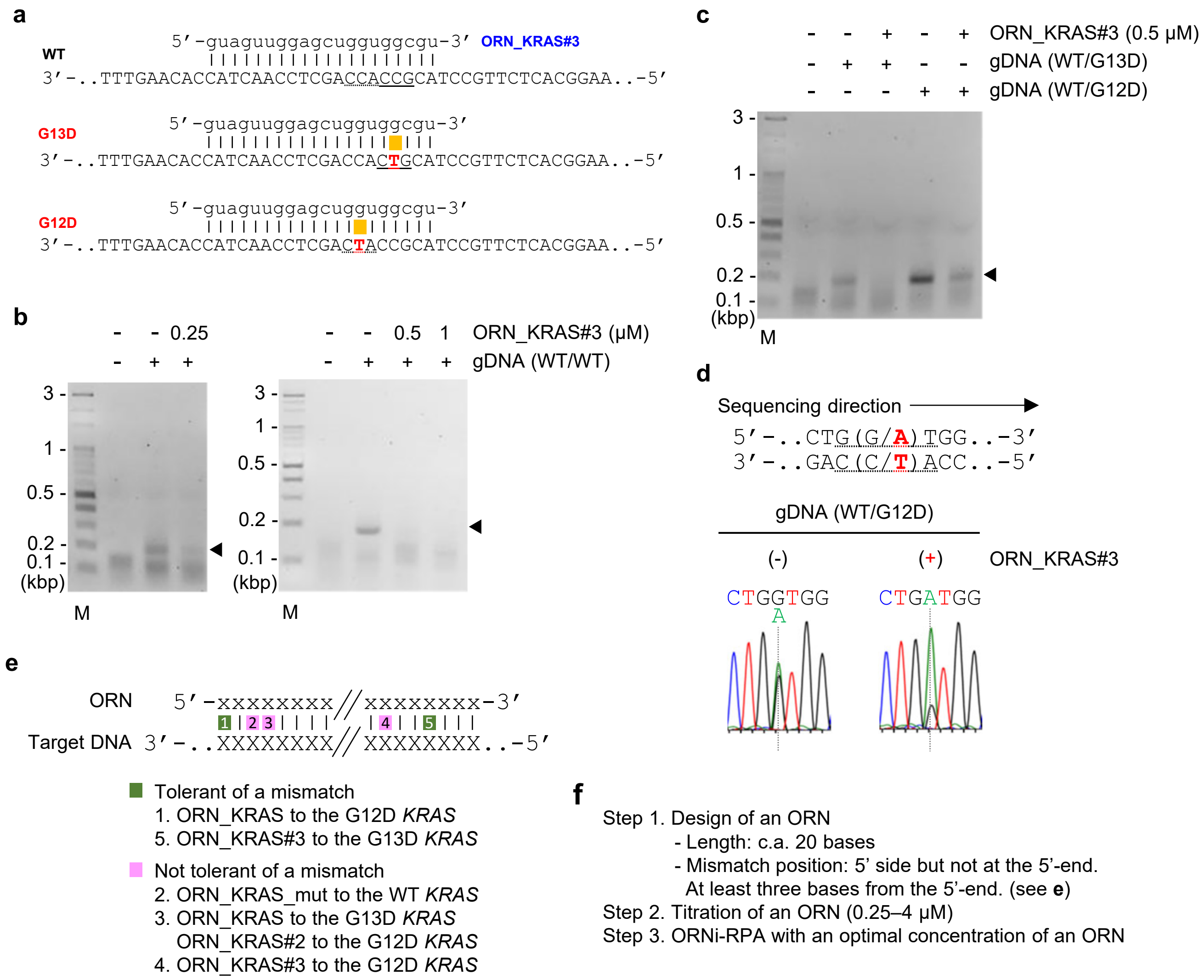

Supplementary Figure 2. Discrimination of a single-nucleotide mutation by ORNi-RPA. (**a**) An ORN examined as a sequence-specific blocking agent and its complementary sequence in the human *KRAS* gene. (**b**) ORNi-RPA with different doses of an ORN. 293T gDNA (WT/WT *KRAS*) was used as a template. M, molecular weight marker. (**c**) Results of ORNi-RPA of HCT116 gDNA (WT/G13D *KRAS*) and a commercially available gDNA (WT/G12D *KRAS*). (**d**) DNA sequencing analysis of ORNi-RPA products. RPA and ORNi-RPA products in **c** were purified and sequenced using a forward primer. (**e**) Summary of tolerance of nucleotide mismatches between ORNs and target DNA. (**f**) A potential step-by-step procedure for ORNi-RPA.

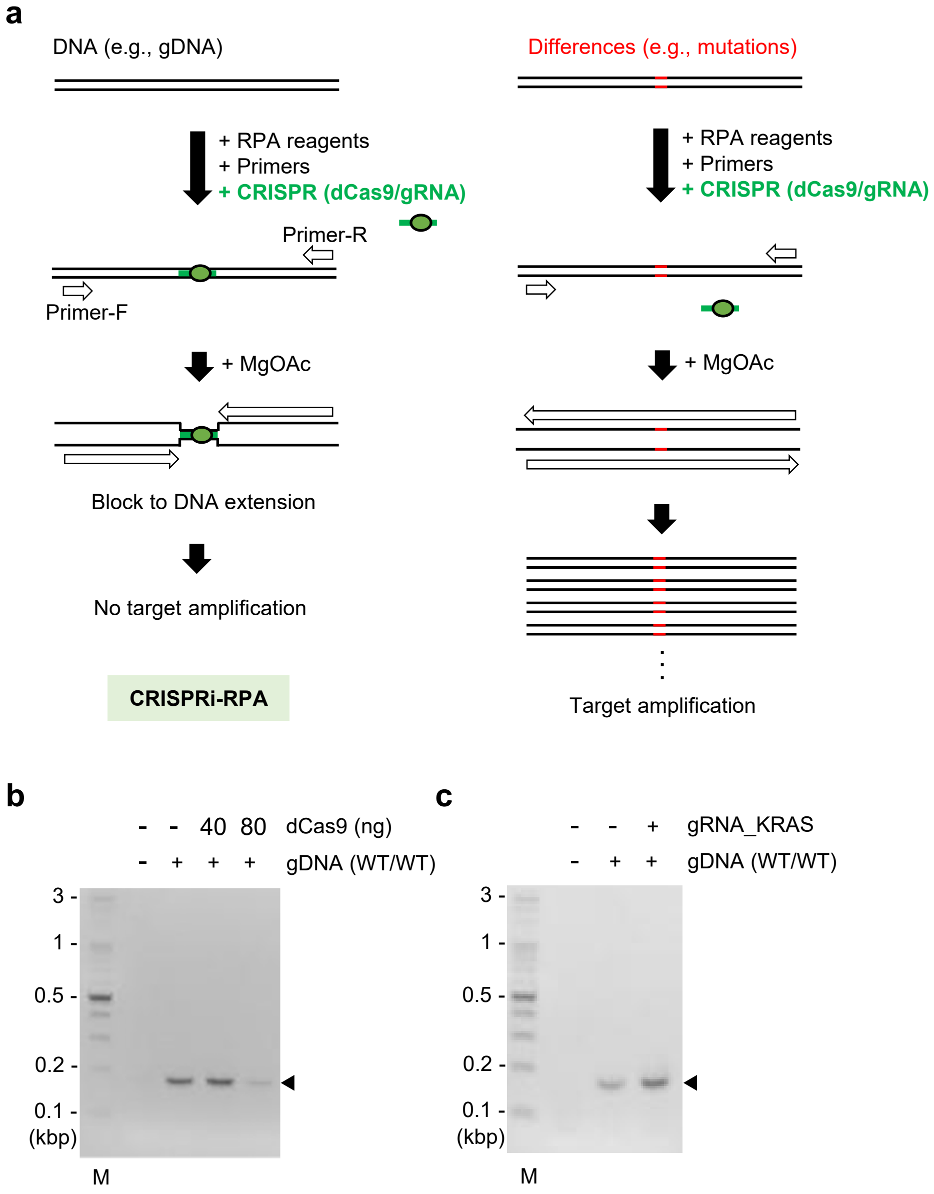

Supplementary Figure 3. Sequence-specific suppression of RPA reactions by CRISPRi. (**a**) Schematic diagram showing sequence-specific suppression of RPA reactions by CRISPRi (CRISPRi-RPA). In CRISPRi-RPA, dCas9 plus gRNA block DNA extension by the DNA polymerase, resulting in the suppression of DNA amplification across a gRNA target sequence. If a gRNA target sequence is mutated (substitution, insertion, or deletion), dCas9 plus gRNA cannot bind to the mutated sequence, which allows DNA amplification to proceed. (**b**) RPA reactions in the presence of only dCas9. The human *KRAS* gene was amplified from 293T gDNA (WT/WT *KRAS*). RPA reactions were not inhibited in the presence of 40 ng of dCas9 so this dose was adopted in all CRISPRi-RPA experiments. M, molecular weight marker. (**c**) RPA reactions in the presence of gRNA alone. The human *KRAS* gene was amplified from 293T gDNA. RPA reactions were not inhibited in the presence of gRNA alone (10 nM in an RPA reaction mixture).

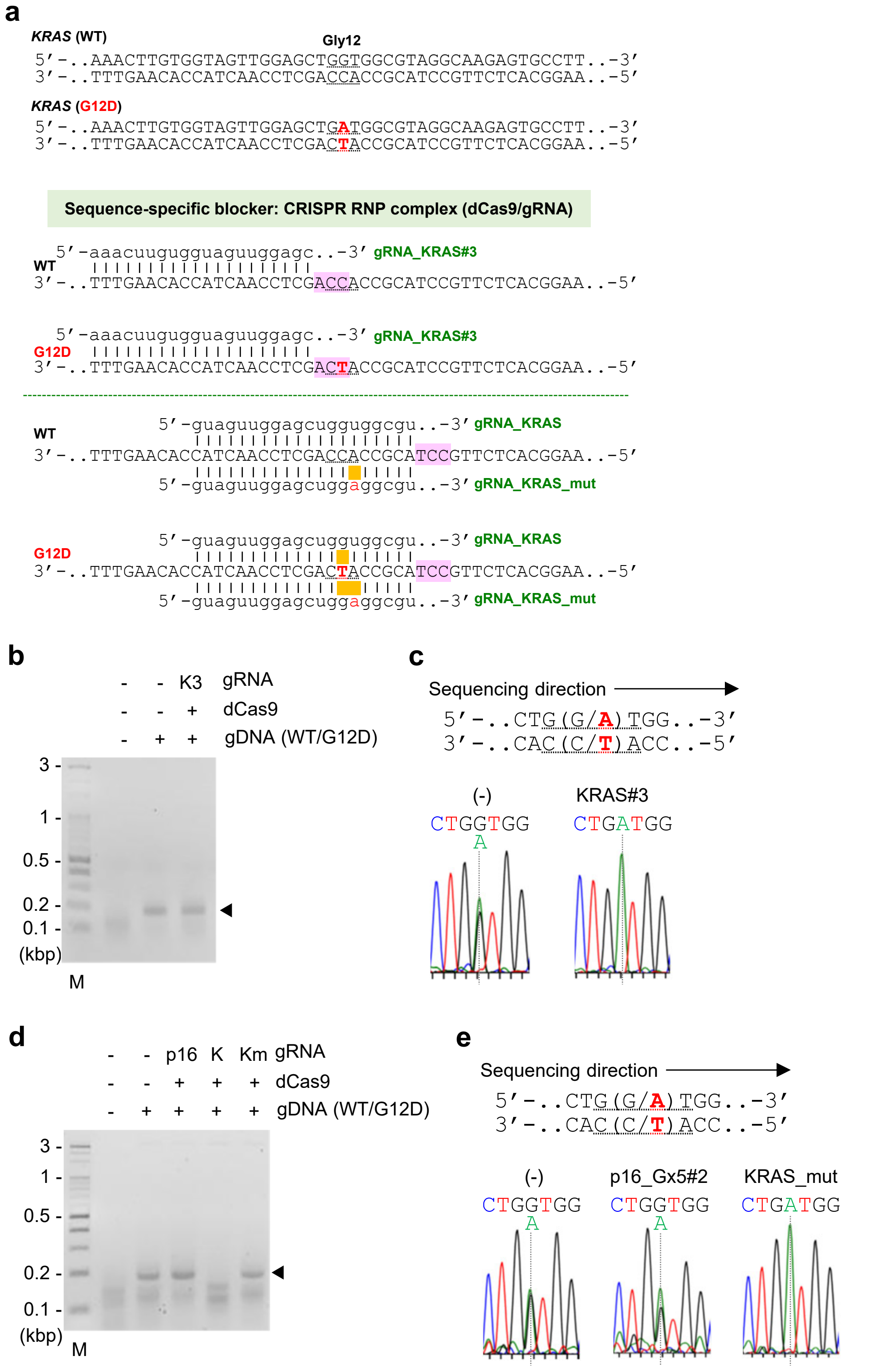

Supplementary Figure 4. Discrimination of the G12D *KRAS* mutation by CRISPRi-RPA. (**a**) gRNAs and their target sequences in the human *KRAS* gene. (**b** and **d**) Results of CRISPRi-RPA of a commercially available gDNA (WT/G12D *KRAS*). Amplified *KRAS* is indicated by an arrowhead. M, molecular weight marker. (**c** and **e**) DNA sequencing analysis of CRISPRi-RPA products. RPA and CRISPRi-RPA products from **b** and **d** were purified and sequenced using a forward primer.

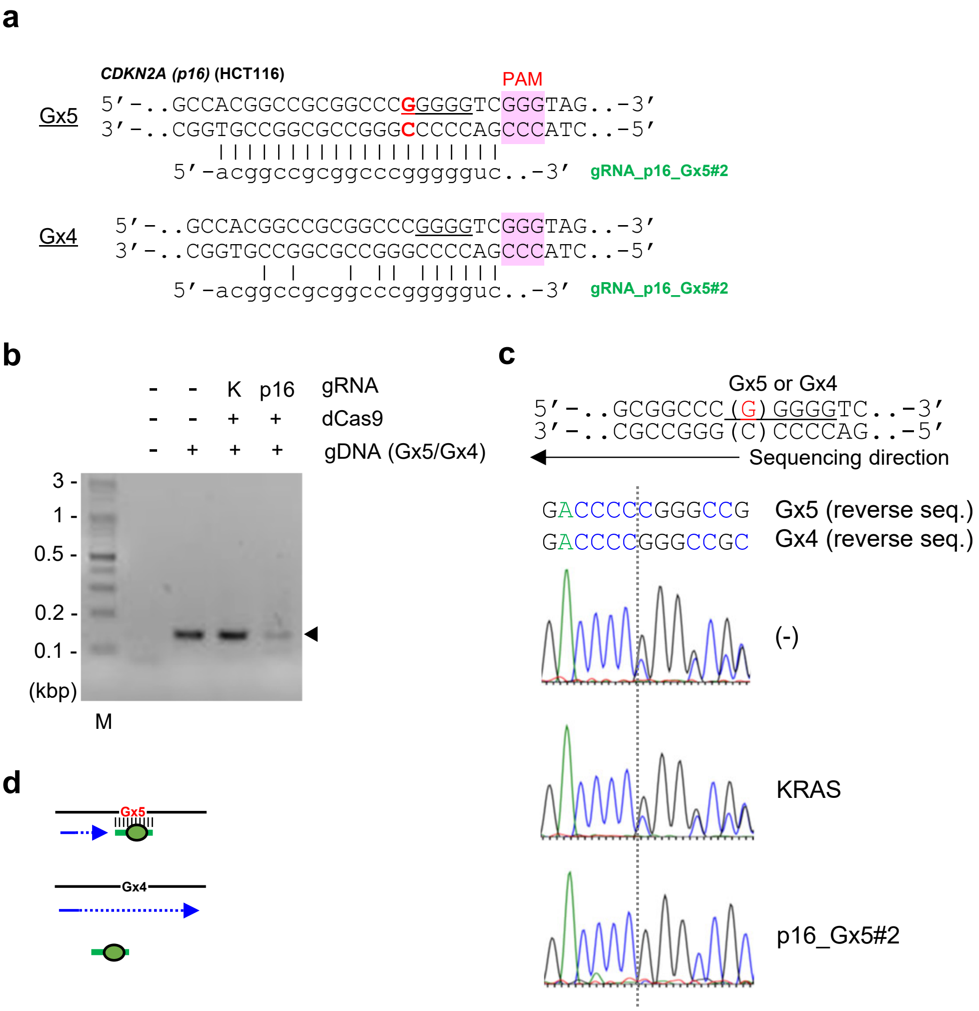

Supplementary Figure 5. Discrimination of a single-nucleotide insertion/deletion by CRISPRi-RPA. (**a**) The target DNA sequence in the *CDKN2A (p16)* gene in HCT116 cells. A single guanine is inserted in the *CDKN2A (p16)* gene in one allele (shown as Gx5). Forward and reverse sequences of the gene are shown. (**b**) Results of CRISPRi-RPA. The *CDKN2A (p16)* gene was amplified from HCT116 gDNA. M, molecular weight marker. (**c**) Results of DNA sequencing analysis. RPA and CRISPRi-RPA products in **b** were purified and sequenced using a reverse primer. (**d**) A model to show how CRISPRi-RPA discriminates single-guanine mutations in the *CDKN2A (p16)* gene.

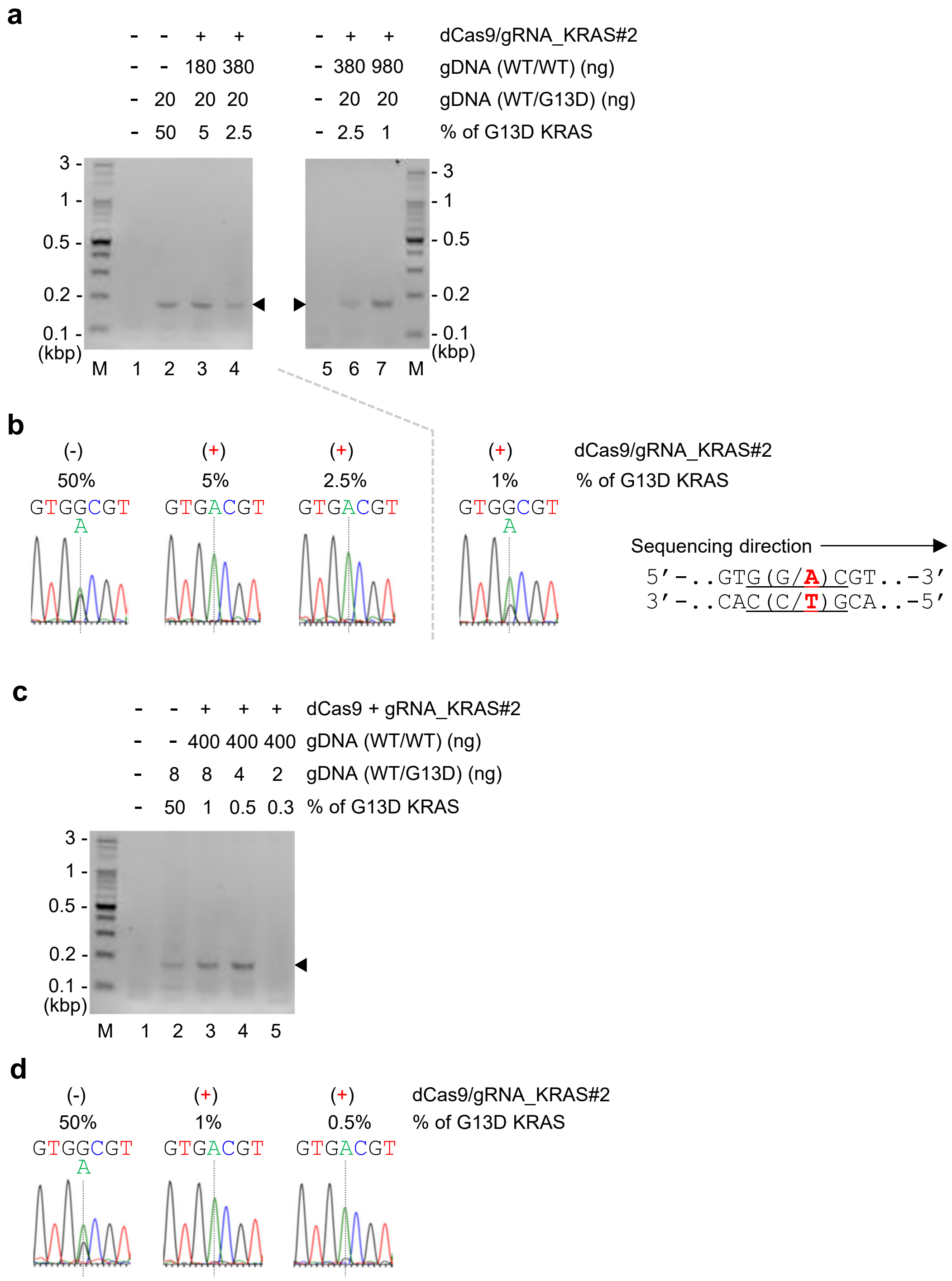

Supplementary Figure 6. Sensitivity of CRISPRi-RPA. (**a** and **c**) Results of CRISPRi-RPA of the human *KRAS* gene. Various doses of 293T gDNA (WT/WT *KRAS*) and HCT116 gDNA (WT/G13D *KRAS*) were mixed so that the G13D *KRAS* accounted for 5–0.3% of the total *KRAS*. These gDNA mixtures were used for RPA in the presence or absence of dCas9/gRNA_KRAS#2. M, molecular weight marker. (**b** and **d**) Results of DNA sequencing analysis of the RPA or CRISPRi-RPA amplicons shown in **a** and **c**.

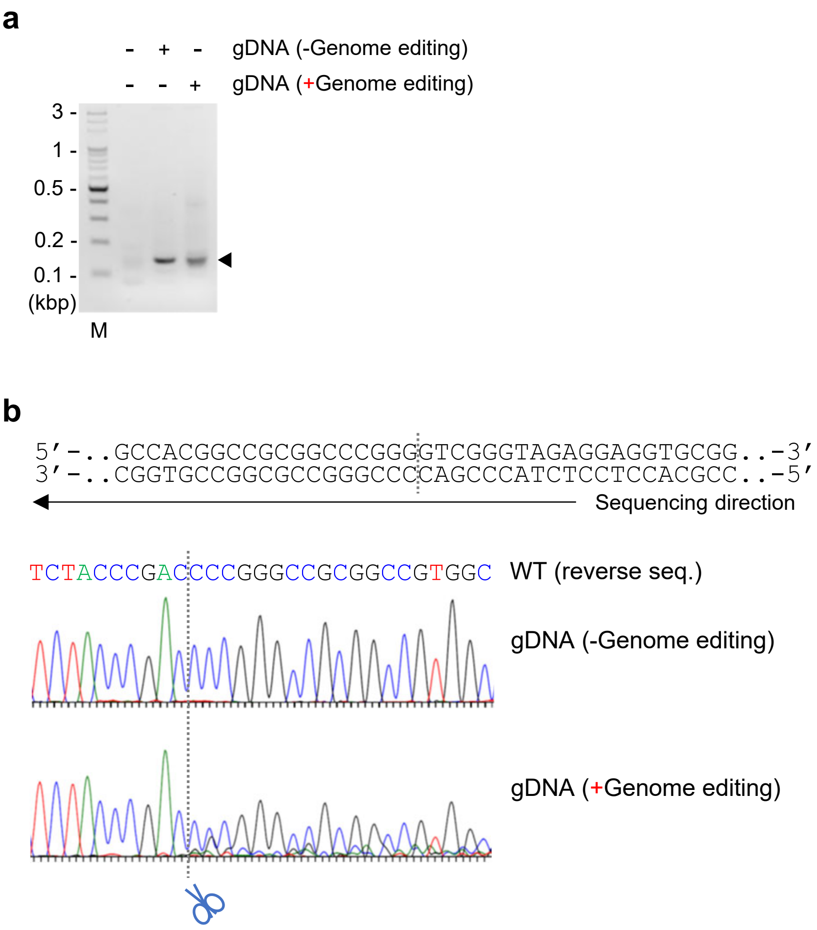

Supplementary Figure 7. RPA with gDNA extracted from genome-edited 293T cells. (**a**) Results of RPA. The human *CDKN2A (p16)* gene was amplified from gDNA extracted from parental and genome-edited 293T cells. M, molecular weight marker. (**b**) Results of DNA sequencing analysis. RPA products in **a** were purified and sequenced using a reverse primer.

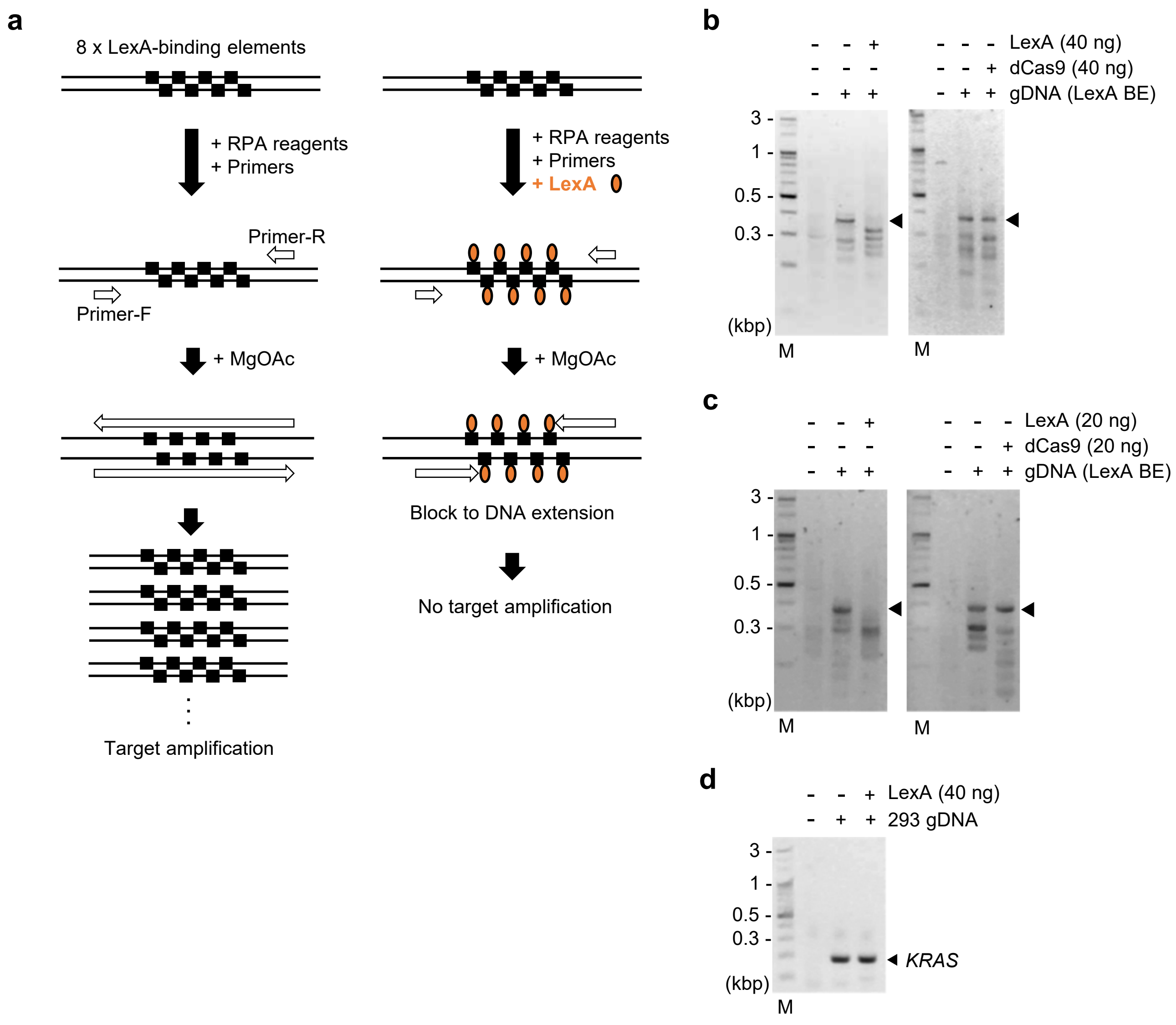

Supplementary Figure 8. Suppression of RPA reactions by a sequence-specific DNA-binding protein. (**a**) Schematic diagram showing sequence-specific suppression of RPA reactions by the bacterial DNA-binding protein LexA. RPA amplifies a DNA sequence including 8 copies of LexA-binding elements (LexA BE). In the presence of LexA, DNA polymerases are blocked by LexA binding to LexA BE, resulting in the suppression of amplification. (**b**–**d**) Results of RPA in the presence of LexA or dCas9. The DNA sequence including 8 copies of LexA BE was amplified from DT40#205-2 gDNA (**b** and **c**). Alternatively, the human *KRAS* gene was amplified as an irrelevant sequence from 293T gDNA (**d**). dCas9 was used as a negative control protein. M, molecular weight marker.

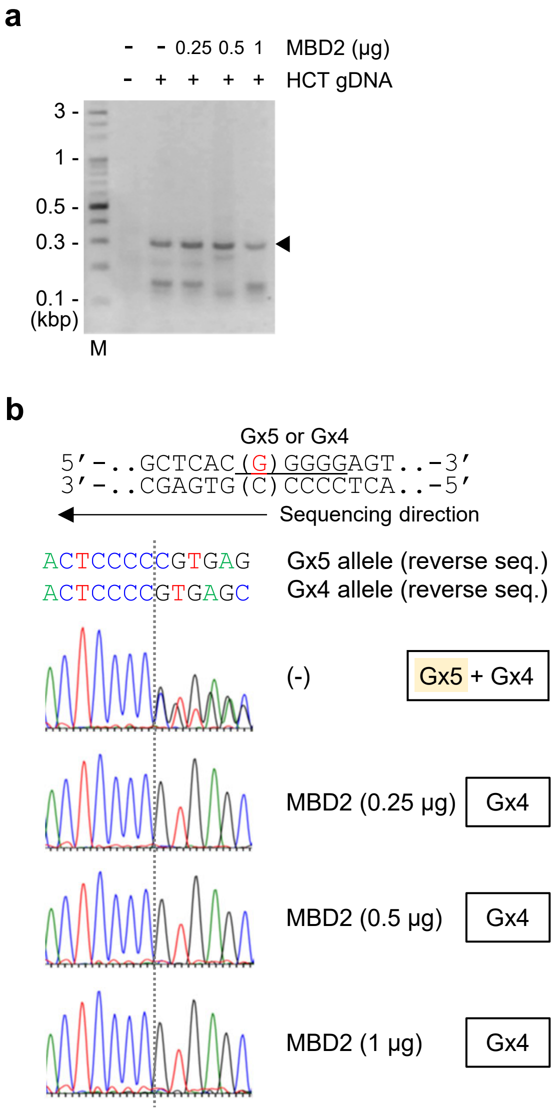

Supplementary Figure 9. MBDi-RPA on the human *CDKN2A (p14ARF)* gene. (**a**) Results of MBDi-RPA with various doses of MBD2 proteins. The human *CDKN2A (p14ARF)* gene was amplified. M, molecular weight marker. (**b**) DNA sequencing analysis of MBDi-RPA products. MBDi-RPA products in **a** were purified and sequenced using a reverse primer.

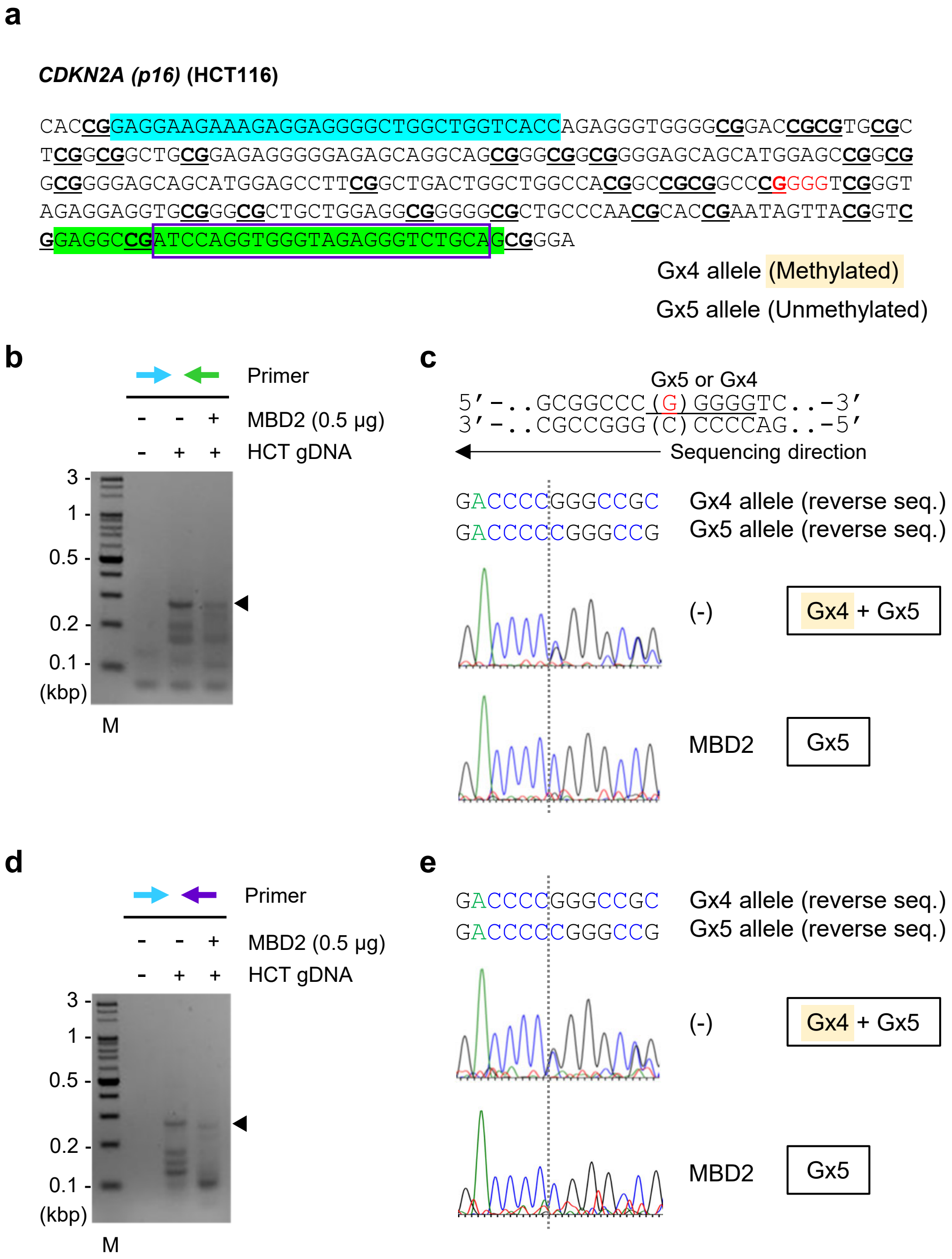

Supplementary Figure 10. MBDi-RPA on the human *CDKN2A (p16)* gene. (**a**) The target sequence and primer positions for the human *CDKN2A (p16)* gene in HCT116. Forward and reverse primer positions are highlighted in blue and green, respectively. Another reverse primer position is indicated by a purple square. (**b** and **d**) Results of MBDi-RPA. M, molecular weight marker. (**c** and **e**) Results of DNA sequencing analysis. RPA or MBDi-RPA amplicons shown in **b** and **d** were sequenced with a reverse primer.

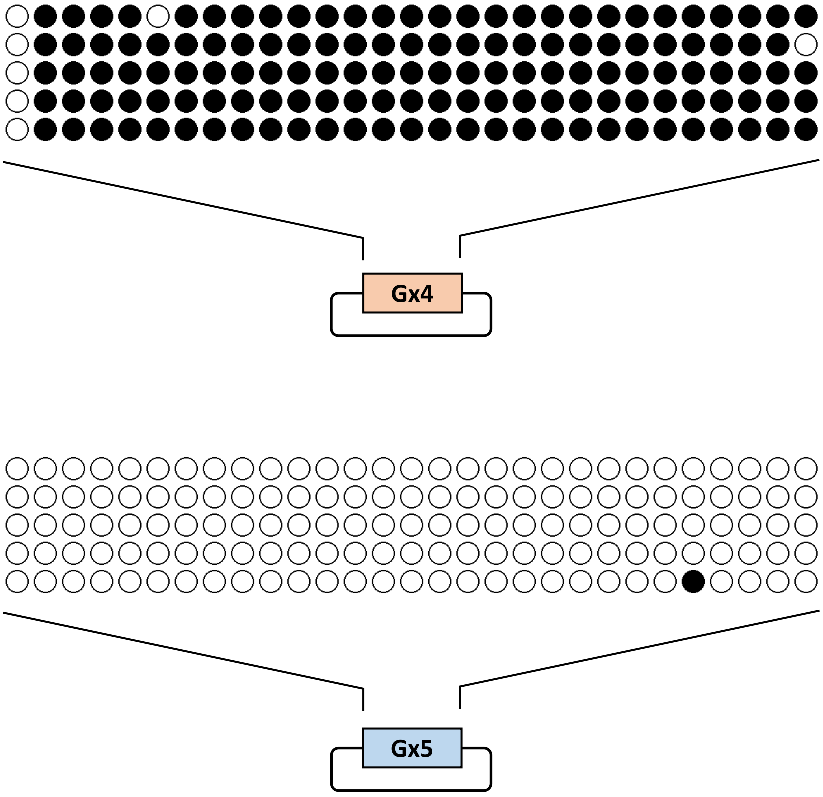

Supplementary Figure 11. Results of bisulfite-sequencing. The human *CDKN2A (p14ARF)* sequence shown in Figure 4b was amplified by PCR and then cloned into plasmids. The plasmid containing the Gx4 sequence was methylated by a CpG-methyltransferase M.SssI *in vitro* while the plasmid containing of the Gx5 sequence was mock-treated. To evaluate CpG-methylation status, the resultant plasmids were subjected to bisulfite-treatment. The *CDKN2A (p14ARF)* sequences were amplified from each plasmid by PCR, cloned, and sequenced. White circles show non-CpG-methylated sites whereas black circles show CpG-methylated sites.

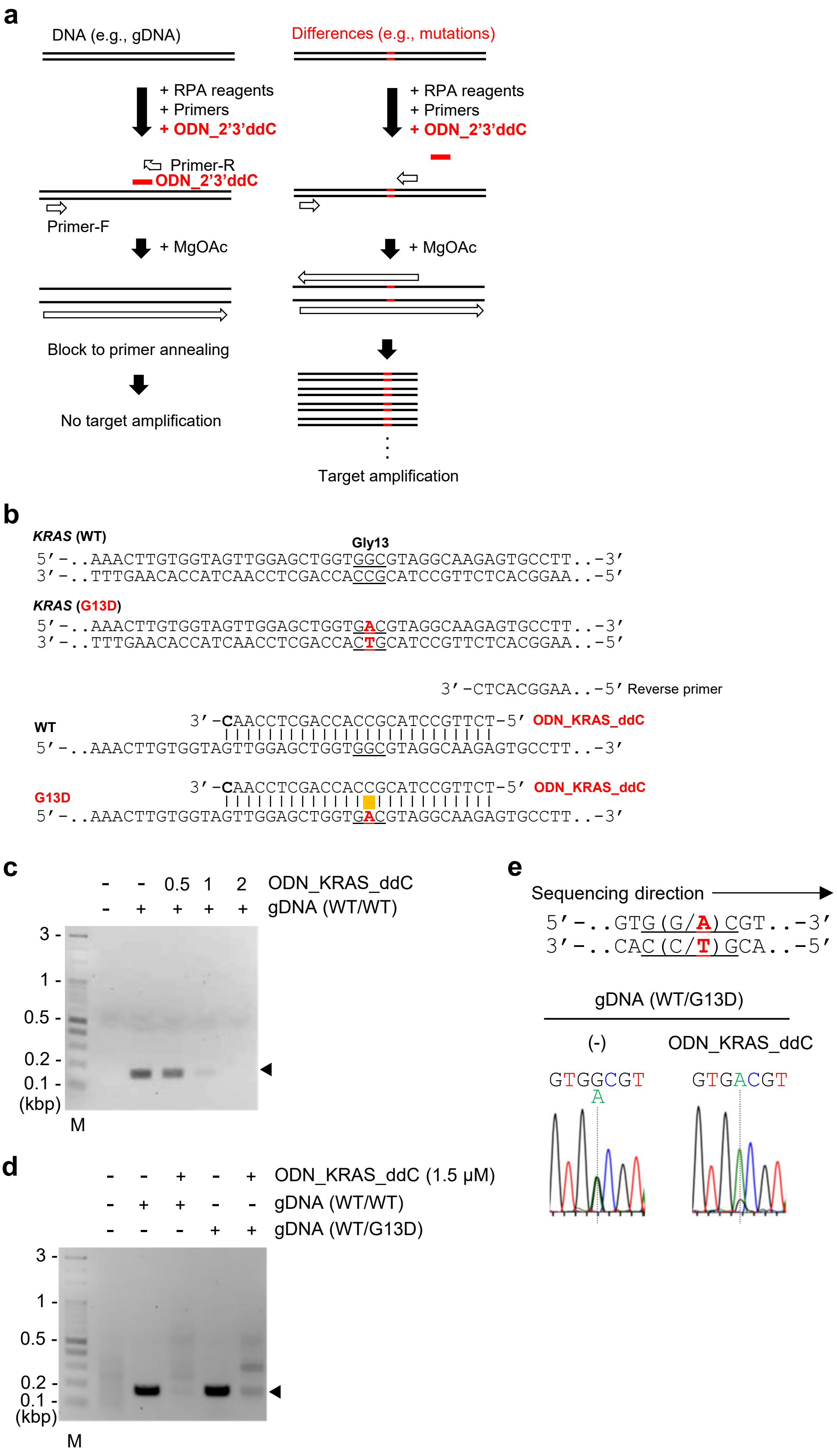

a
DNA (e.g., gDNA)
Differences (e.g., mutations)
+ RPA reagents
+ Primers
+ ODN_2'3'ddC
Primer-R
ODN_2'3'ddC
Primer-F
+ MgOAc
Block to primer annealing
No target amplification
Target amplification
b
KRAS (WT)
Gly13
5'-..AAACTTGTGGTAGTTGGAGCTGGTGGCGTAGGCAAGAGTGCCTT..-3'
3'-..TTTGAACACCATCAACCTCGACCACCGCATCCGTTCTCACGGAA..-5'
KRAS (G13D)
5'-..AAACTTGTGGTAGTTGGAGCTGGTGACGTAGGCAAGAGTGCCTT..-3'
3'-..TTTGAACACCATCAACCTCGACCACTGCATCCGTTCTCACGGAA..-5'
3'-CTCACGGAA..-5' Reverse primer
WT
3'-CAACCTCGACCACCGCATCCGTTCT-5' ODN_KRAS_ddC
5'-..AAACTTGTGGTAGTTGGAGCTGGTGGCGTAGGCAAGAGTGCCTT..-3'
G13D
3'-CAACCTCGACCACCGCATCCGTTCT-5' ODN_KRAS_ddC
5'-..AAACTTGTGGTAGTTGGAGCTGGTGACGTAGGCAAGAGTGCCTT..-3'
c
- - 0.5 1 2 ODN_KRAS_ddC
- + + + + gDNA (WT/WT)
3
1
0.5
0.2
0.1
(kbp)
M
d
- - + - + ODN_KRAS_ddC (1.5 μM)
- + + - - gDNA (WT/WT)
- - - + + gDNA (WT/G13D)
3
1
0.5
0.2
0.1
(kbp)
M
e
Sequencing direction
5'-..GTG(G/A)CGT..-3'
3'-..CAC(C/T)GCA..-5'
gDNA (WT/G13D)
(-)
ODN_KRAS_ddC
GTGGCGT
A
GTGACGT

Supplementary Figure 12. Discrimination of the G13D *KRAS* mutation by blocking RPA with a 3'-modified ODN. (**a**) Schematic diagram showing blocking RPA with a 3'-modified ODN. In the presence of an ODN modified with 2'3'ddC at the 3'-end, annealing of a primer is inhibited, resulting in suppression of DNA amplification. If the target sequence is mutated, it cannot be recognized by the ODN and DNA amplification proceeds uninhibited (allowing the detection of a DNA mutation). (**b**) A 3'-modified ODN and its complementary sequences in the human *KRAS* gene. (**c**) Blocking RPA with various doses of a 3'-modified ODN. 293T gDNA (WT/WT *KRAS*) was used as a template. M, molecular weight marker. (**d**) Results of blocking RPA of 293T gDNA (WT/WT *KRAS*) and HCT116 gDNA (WT/G13D *KRAS*). (**e**) DNA sequencing analysis of blocking RPA products. RPA and blocking RPA products in **d** were purified and sequenced using a forward primer.

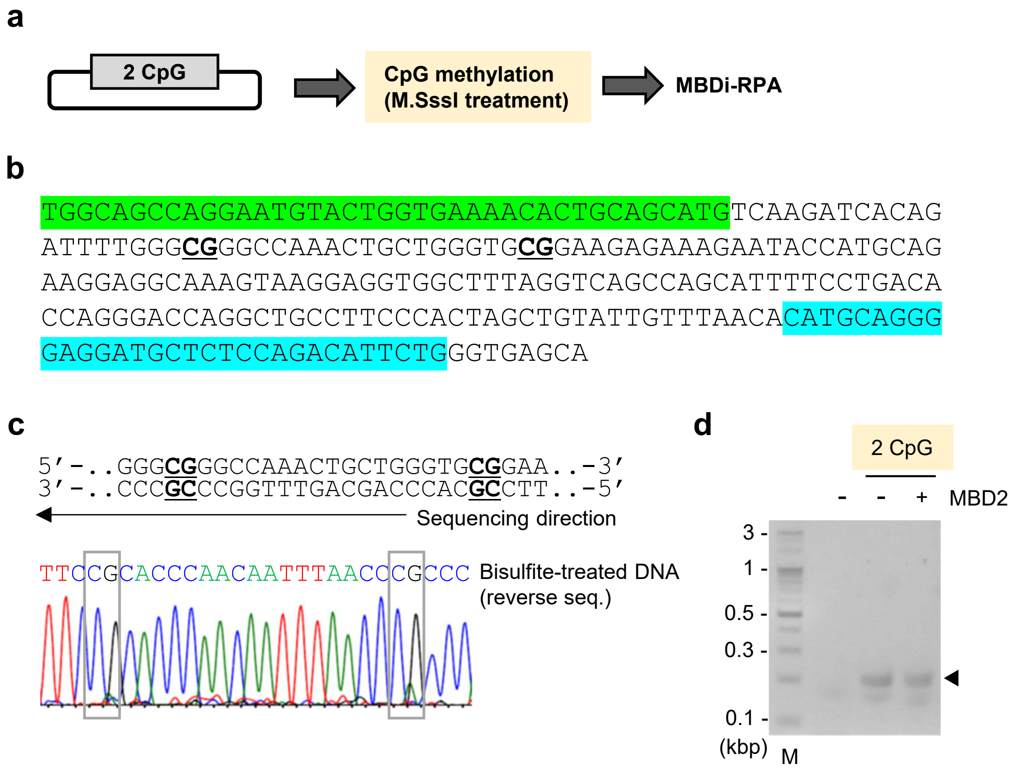

Supplementary Figure 13. MBDi-RPA with template DNA containing two CpG sites. (**a**) Schematic diagram showing *in vitro* CpG methylation followed by MBDi-RPA. A human *EGFR* sequence was cloned into a plasmid, CpG-methylated, and used for MBDi-RPA. (**b**) The human *EGFR* sequence cloned in a plasmid. Forward and reverse primer positions are shown in blue and green, respectively. (**c**) Results of bisulfite-sequencing analysis. To confirm *in vitro* methylation, the CpG-methylated plasmid was subjected to bisulfite treatment followed by PCR amplification. The amplicon was directly sequenced with a reverse primer. The CpG sites are shown in gray squares. (**d**) Results of MBDi-RPA. M, molecular weight marker.
